# Roles of TREM2 in the Pathological Mechanism and the Therapeutic Strategies of Alzheimer’s Disease

**DOI:** 10.14283/jpad.2024.164

**Published:** 2024-09-10

**Authors:** M. Lin, J.-X. Yu, W.-X. Zhang, F.-X. Lao, Han-Chang Huang

**Affiliations:** 1 Beijing Key Laboratory of Bioactive Substances and Functional Foods, Beijing Union University, Beijing, 100023 China; 2 Key Laboratory of Natural Products Development and Innovative Drug Research, Beijing Union University, Beijing, 100023 China; 3 No. 18, Fatou third block, Chaoyang District, Beijing, China

**Keywords:** Alzheimer’s disease (AD), triggering receptor expressed on myeloid cells 2 (TREM2), microglia, Aβ, monoclonal antibody

## Abstract

Alzheimer’s disease (AD) is an age-related degenerative disease, which is characteristic by the deposition of senile plaques (SP) outside the cells, the neurofibrillary tangles (NFTs) inside the neurons, and the loss of synapse and neurons. Neuroinflammation may play an important role in the pathogenesis of AD. Microglia are the immune cells in the central nervous system. However, microglia might become disease-related microglia (DAMs) when stimulated by the external environment. DAMs have been shown to be involved in a series of events of AD development including Aβ accumulation and tau phosphorylation. The triggering receptor expressed on myeloid cells 2 (TREM2) is a transmembrane receptor that is mainly expressed by microglia in the central nervous system (CNS). TREM2 plays an important role in the physiological function of microglia, and the dyshomeostasis of TREM2 is related to the development of late-onset AD. This article summarized the latest advances in TREM2 biology and its impact on the roles of microglia in AD development, with a particular emphasis on the structure, ligands, signal transduction, and the agonistic antibodies of TREM2 for AD treatment. We further discussed the survival, migration, phagocytosis, inflammation, and cellular metabolism of microglia, as well as the role of sTREM2 in neuroprotection and as a biomarker for AD. It provides a reference for further research on the molecular mechanism of microglial TREM2 in the occurrence and development of AD and on the therapeutic strategies targeted on the microglial TREM2.

**A**lzheimer’s disease (AD) is an age-related degenerative disease. The global number of populations with AD dementia is about 32.0 million and the prevalence of AD will more than triple by 2050 all over the world (1, 2). In China, according to the data released by the seventh Chinese National Census in 2021 and China Alzheimer’s Disease Report 2021, there are 264 million people aged 60 over, of which AD patients account for 3.72% (3). At present, there are less substantial breakthrough in the research on the pathogenesis of AD and the development of therapeutic drugs. The existing drugs for treating AD have very limited therapeutic effects and can only temporarily alleviate AD symptoms..

The pathological features of AD mainly show the extracellular depositions of senile plaques (SPs), intracellular neurofibrillary tangles (NFTs), and neuronal and synaptic loss (4, 5). Senile plaques mainly contain amyloid-β (Aβ) peptides and are surrounded by active microglia, while neurofibrillary tangles are made up of paired-helical filaments caused by hyperphosphorylated tau protein. Additionally, the AD development is accompanied by the proliferation of microglia and astrocytes, and activated microglia and astrocytes are present around senile plaques (6, 7).

Microglia, the resident macrophages of the brain and spinal cord, are involved in the development of the nervous central system (CNS) and maintain a homogeneous cell population in the brain. In pathological conditions, however, microglia play important roles on the pathogenesis of many neurodegenerative diseases. Recently, the disease-associated microglia (DAM) have been identified by single-cell RNA sequencing (scRNA-seq) analysis, and DAM are associated with neurodegenerative diseases (8). The AD risk genes including TREM2 (triggering receptor expressed on myeloid cells 2) are highly expressed in microglia, indicating that microglia dysfunction might be one of the main culprits in AD development (6). Therefore, there may be a close association among microglia, TREM2 and AD.

In recent years, the research community has shifted its attention to the key role of myeloid cells in various pathologies. TREM2 is an innate immune receptor expressed on myeloid lineage cells. The TREM2 gene was originally found in mouse macrophages and monocyte-derived dendritic cells by the Swiss scientist J.M. Gollie and S.D. Cohen (9). In AD pathology, the interaction between TREM2 and DNAX activation protein 12 (DAP12) can activate the pathways involved in microglial activation and phagocytosis. These pathways play an important role in microglial function including migration and phagocytosis, brain lipid metabolism and anti-inflammation, and neuronal apoptosis (10). In additional, 46 kinds of TREM2 mutants associated with AD, including R47H, R62H, Q33X, T66M, and Y38C, have been found. These mutants increase the risk of AD development (11).

Notably, soluble TREM2 (sTREM2), an extracellular domain fragment of TREM2, also plays an important role in the pathological process of AD. On the one hand, sTREM2, as a short peptide with important biological functions, can promote the proliferation and migration of microglia around amyloid plaques, reduce the amyloid plaque burden, and improve the learning and memory ability of AD mice (12). On the other hand, sTREM2 can be detected in cerebrospinal fluid (CSF). The increase of the CSF sTREM2 in AD patients often precedes the decline in cognition, and the level of sTREM2 is positively correlated with the level of hyperphosphorylated tau protein in the cerebrospinal fluid (13, 14). In general, the accumulated evidence highlights the potential of sTREM2 as a biomarker and therapeutic target in AD (15, 16).

This review summarized the recent research progress on the roles and mechanisms of TREM2 in AD pathology and the therapeutic strategies for AD targeted on TREM2.

## TREM2

### Structure of TREM2

**Figure 1:**
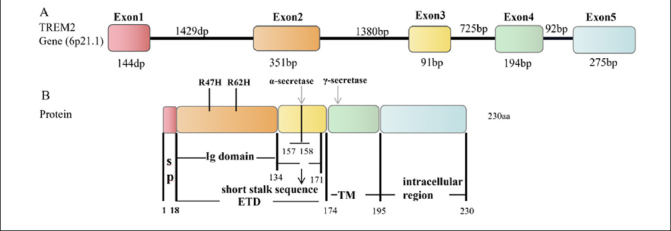
Structure of the human TREM2 gene and protein A: TREM2 gene, containing the exon (number box) and intron (line) sequences. B: TREM2 protein structure, including the signal peptide (SP) (1–18 aa), immunoglobulin Ig domain (19–134 aa), short stalk sequence (135–171 aa) in the extracellular region ETD (19–174 aa), and transmembrane (TM) region (175–195 aa) and intracellular region (196-230 aa). Arrows indicate the α-secretase and γ-secretase cleavage sites, respectively. The α-secretase cleaves TREM2 at the His157-Ser158 site. Two common AD-related variants, R47H and R62H, are present in the Ig domain, and the Ig domain may be involved in a key function mediated by TREM2 signaling.

The TREM2 gene is located on human chromosome 6, and it contains five exons, producing three transcripts by alternative splicing of pre-mRNA (ENST00000373113, ENST00000373122, ENST00000338469) (17). ENST00000373113 contains five exons and encodes a full-length protein of 230 amino acid residues(18). The TREM2 protein structure includes the extracellular type V immunoglobulin (Ig) domain (19–134 aa), the transmembrane domain (175–195 aa), and the intracellular domain (196–230 aa) (19, 20) (Fig. 1). The cell signaling induced by TREM2 is mediated by these three regions, in which the extracellular domain (19–174 aa) binds extracellular ligand and the intracellular structure binds intermediate signaling proteins. TREM2 is mainly expression in microglia in the hippocampus, spinal cord, and white matter (21). TREM2 is essential for the physiologic function of microglia.

### sTREM2

#### Generation of the sTREM2

The fragment of TREM2’s extracellular domain, also called soluble TREM2 (sTREM2), is found in human cerebrospinal fluid (CSF) and serum. The sTREM2 may be generated via two different mechanisms: (1) proteolytic shedding of the TREM2 ectodomain and (2) alternative splicing of the TREM2 pre-mRNA (22). On the one hand, TREM2 would be broken at the peptide bond between histidine 157 and serine 158 (His157-Ser158) in the presence of a disintegrin and metalloproteinase 10 (ADAM10) or ADAM17 to shed the extracellular domain of TREM2 (23). Additionally, Meprin β, a zinc metalloproteinase that belongs to the astacin family of proteases, can cleave TREM2 at the location between arginine 136 and aspartic acid (Arg136-Asp137) to produce sTREM2 (24). On the other hand, the splicing variant of sTREM2 is produced when pre-mRNA of TREM2 was alternatively processed after transcription, and the splicing variant is translated into sTREM2, which lacks the transmembranal and intracellular domains (25). Up to 25% of the sTREM2 may be produced by the expression of splicing variations but not by the cleavage of TREM2 (26).

Mutations within the TREM2 gene might significantly affect the sTREM2 production, and the specific variants might cause the translated protein to misfold and remain the protein in the endoplasmic reticulum, thereby reducing sTREM2 secretion (27).

#### The CSF sTREM2 is related to AD development

The level of sTREM2 is associated with AD progress, and it is increased in the early course of AD. It seems that the CSF sTREM2 not only can distinguish AD individuals from healthy individuals but also can monitor the AD development from early stage to mild cognitive impairment (MCI) (28). In recent study, it is suggested that higher level of CSF sTREM2 is associated with higher CSF p-tau or total tau and amyloid-β levels (29) and the increase rate is significantly upregulated in patients with both Aβ and tau pathologies (30). Therefore, the sTREM2 may be used as a diagnostic biomarker for early detection of AD. Meanwhile, the sTREM2 might also serve as a potential target for AD treatment.

However, the physiological function of sTREM2 remains poorly understood. One alternative hypothesis is that sTREM2 might act as a decoy receptor that competes for the endogenous ligands with membrane-bound TREM2. The sTREM2 might counter AD development by interfering with Aβ and tau pathologies. In the 5×FAD mouse model, it was shown that sTREM2 reduced Aβ accumulation and improved behavioral disorders (31). Moreover, overexpress of sTREM2 in APP mice diminished the plaque burden and significantly reversed the impairments in long-term potentiation (LTP) and spatial memory deficits (32). Additionally, the sTREM2 might reduce tau phosphorylation to counter AD development. The sTREM2 can induce phosphorylation of RhoA at Ser188 when binding to transglutaminase 2 (TG2), thus inhibiting the RhoA/ROCK (Rho-associated coiled-coil kinase)/Glycogen synthase kinase 3β (GSK3β) pathway as well as tau phosphorylation (33).

### TREM2 ligands and downstream signaling pathways

**Figure 2:**
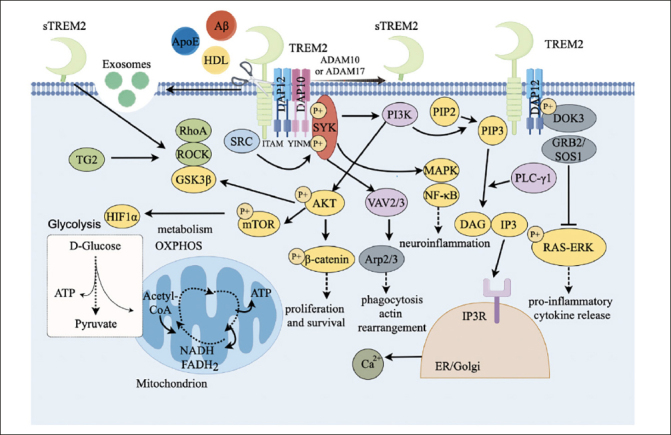
TREM2 signaling pathway TREM2=myeloid cell trigger receptor 2; sTREM2=soluble TREM2; ApoE=apolipoprotein E; HDL=high-density lipoprotein; LPS=lipopolysaccharide; DAP12=DNAX activator protein 12; DAP10=DNAX-activated protein 10; ITAM=immune receptor tyrosine activation motif; SYK=splenic tyrosine kinase; PI3K=Phosphatidylinositol 3-kinase; AKT= serine / threonine protein kinase; MTOR=target of rapamycin; PIP3=3, 4,5-phosphatidylinositol triphosphate; P1P2=phosphatidylinositol-4, 5-bisphosphate; PLC-γ=PLC-γ; phospholipase; IP3=1, 4,5-inositol triphosphate; DAG=diglyceride; NF-κB=nuclear factor κB; MAPK=mitogen-activated protein kinase; hypoxia inducible factor-1α=HIF1α; Vav guanine nucleotide exchange factor 2/3=VAV2/3; GRB2=growth factor receptor-bound protein 2; SOS1=son of sevenless 1; RAS=rat sarcoma; ERK=extracellular signal-regulated kinase; Arp2=actin-related protein 2; Arp3=actin-related protein 3; β-catenin=beta-catenin; SRC= proto-oncogene c-Src protein tyrosine kinase; GSK3β=glycogen synthase kinase 3β; TG2=transglutaminase 2; ROCK=Rho-associated coiled-coil-containing protein kinase; ATP=Adenosine triphosphate; NADH=nicotinamide adenine dinucleotide Hydrogen; FADH_2_=flavin adenine dinucleotide reduced; ADAM10/17=a disintegrin and metalloprotease domain-containing protein 10/17; DOK3=downstream of kinase 3.

Multiple ligands can recognize and bind TREM2 at the cell membrane to activate TREM2 cascade signaling, thereby affecting microglial function (Fig. 2). The analysis of lipid-binding arrays with recombinant TREM2 revealed that TREM2 binds to anionic and zwitterionic lipids (34). These ligands include anionic carbohydrates, anionic bacterial products, apoprotein E (ApoE) and various phospholipids including aminophospholipid (35), phosphatidylserine (36), and glycolipids. TREM2 can directly interact with pathological Aβ oligomers and lipoproteins, and TREM2 together with Aβ was found in the senile plaques. Research has shown that the affinity of TREM2 extracellular domain for binding to Aβ varies among different amyloid protein structures, with the highest affinity for binding to Aβ oligomers (37).

TREM2 affects the function of microglia through multiple signaling pathways in immune regulation and neurodegenerative diseases (Fig. 2). TREM2 can be activated when microglia were induced to upregulate expression of ApoE, leading to the upregulation of lipoprotein-lipase (LPL) expression (38). In turn, the interaction of ApoE with Aβ, lipids, and heparan sulfate affects the phagocytosis of microglia (39). TREM2 binds to the adapter protein DNAX activation protein 12 (DAP12) or DAP10 through amino acid residues with opposite charges in its transmembrane domain (40). DAP12 contains immune receptor tyrosine-based activation motif (ITAM) in the cytosolic region (19, 41). Unlike DAP12, DNAX activation protein 10 (DAP10) contains a short internal cytoplasmic structure, which contains a special tyrosine motif YINM.

After TREM2 binds to the ligand to form a ligand-receptor complex, the ITAM is phosphorylated and then activates the downstream spleen tyrosine kinase (SYK), which is mediated by proto-oncogene c-Src protein tyrosine kinase (SRC). SYK can recruit and activate PI3K, leading to the conversion of phosphatidylinositol-4,5-bisphosphate (PIP2) to phosphatidylinositol-3,4,5-trisphosphate (PIP3). Further, PIP3 is hydrolyzed by phospholipase C-γ (PLC-γ) to generate inositol-3,4,5-trisphosphate (IP3) as well as diacylglycerol (DAG) (42). When binds to IP3 receptor (IP3R) in endoplasmic reticulum (ER) / Golgi membrane, IP3 promotes the release of calcium from ER storage (43). Vav guanine nucleotide exchange factor 2/3 (VAV2/3) complex is also activated by SYK and further induces the organization of Arp2/3 complex to promote rearrangement of actin cytoskeleton with Ca^2+^ (44, 45), affecting microglial phagocytosis. In additional, TREM2 also promotes cell survival and proliferation through PI3K-protein kinase B/serine-threonine specific kinase (AKT)-GSK3β pathway (46).

SYK also can phosphorylate the downstream mitogen-activated protein kinase (MAPK), leading to extracellular-signal regulated kinase (ERK) and AKT phosphorylation (47). MAPK is the central regulator of inflammatory response, and it might activate nuclear factor kappa B (NF-κB) to produce pro-inflammatory cytokines (48). DAG is a key factor involved in the initiation of the nuclear factor kappa B (NF-kB) and the MAPK pathway (49). TREM2 inhibits the MAPK/NF-κB pathway, thereby suppressing the production of pro-inflammatory cytokines and neuroinflammation.

SYK can further enable the activation of PI3K-AKT-mTOR signaling pathway and β-catenin, subsequently promote cell survival (50), or inhibit Toll-like receptor (TLR) to induce the production of inflammatory cytokines (51). In additional, when TREM2 binds to the ligands, DAP12 is recruited to interact with downstream of kinase 3 (DOK3), which further binds to growth factor receptor bound protein 2 (GRB2) / son of sevenless 1 (SOS1) complex to inhibit the RAS-ERK pathway, suppressing pro-inflammatory cytokines production (52).

Additionally, the mTOR activates hypoxia inducible factor-1α (HIF1α), which stimulates glycolysis-related genes, thus regulating cellular metabolisms including lipid metabolism and glycometabolism (53, 54), and the dyshomeostatic metabolisms might result in autophagy.

Therefore, it is important to understand the structure and ligands of TREM2, signal transduction, and functional transition from neuroprotective microglia to disease-associated microglia mediated by TREM2 in AD development.

## TREM2 and disease-associated microglia (DAM)

### Gene expression of DAM

In 2017, Keren-Shaulet al (8) found a novel type of microglial, disease-associated microglia (DAM), which is present around Aβ plaques. DAM not only expresses typical microglial markers, such as Iba1, Cst3, and Hexb, but also shows significant changes in the expression of some other genes (3, 55). Of these genes, MS4A6A, BIN1, PLCG2, PICALM, and CD33 are downregulated but the genes involved in lysosomes, phagocytes, and lipid metabolism pathways, such as ApoE, CTSD, LPL, TYROBP, and TREM2, are upregulated.

### Two phases in which the DAM is activated

Activation of the DAM occurs in two phases: TREM2-independent phase and TREM2-dependent phase. By the knockout and knock-in mice models (TREM2^−/−^ and TREM2^+/+^ mice) Deczkowska et al (56) found that DAM is initially activated in a TREM2-independent manner, involving the activation of ApoE and DAP12 genes. Subsequently the activation of TREM2-dependent program is induced, resulting in the elevation of phagocytic pathway and lipid metabolic processes (56).

Microglia have a practical sense system, named neurodegeneration-associated molecular patterns (NAMPs)), to response to neuronal injury. NAMPs are lipids present in the central nervous system and it can be recognized by TREM2. These lipids of NAMPs can trigger the conversion of microglia to DAM. During demyelination, TREM2 regulates several signal pathways related to lipid metabolism in microglia. A study has found that after chronic demyelination in TREM2^+/+^ and TREM2^+/−^ microglia, the lysosomal genes Ctsb, Ctsd, Ctsz, and the lipid metabolism genes ApoE, Lpl, are significantly upregulated, while P2ry12 gene, which is related to stable microglia, is significantly downregulated in microglia (57). This suggests that TREM2 may be associated with phospholipids, myelin metabolism, and transition of microglia to DAM.

### Effect of DAM on Alzheimer’s disease

In the early stages of AD, DAM inhibits the spread of Aβ plaques by mediating the formation of microglial barriers through TREM2, thereby preventing neuronal damage from Aβ plaques. Therefore, DAM plays a neuroprotective role in the early stage of AD. However, in the late stage of AD, there is a stable stage of tau neurofilaments and Aβ sedimentation. Neuronal death is increased and the inflammatory responses induced by DAM is also increased in the process of clearing the dead neurons. In turn, more neuronal death is resulted and there is a malignant cycle of clearing the dead neurons and inflammatory responses. Therefore, DAM plays a role in neuronal damage in late stage of AD. Alternatively, inhibition of DAM autophagy might cause its detachment from Aβ plaques and aggravates AD neuropathological features.

From a mechanistic perspective, autophagy deficiency might decrease cell proliferation of aging-associated microglia and increase p21 expression, neuronal malnutrition morphology, and secretion of senescence-related factors, such as catalase (Cat), colony-Stimulating Factor 1 (Csf1), complement C3 (C3), and extended Synaptotagmin 1 (Esyt1) (58). SAYED F A et al (59) carried out a study of single-RNA sequencing of brain tissue from 46 AD patients containing the R47H mutation of TREM2. They revealed a subset of microglia enriched with R47H mutation of TREM2. TREM2 increases the release of pro-inflammatory cytokine, activation of AKT signaling, and elevation of a subset of DAM signaling. Inhibiting AKT with MK-2206 can reverse the transcriptome changes of primary microglia, reducing pro-inflammatory cytokine production and synaptic toxicity in AD mice of tauopathies (59). However, further investigations are needed to clarify the specific factors that trigger conversion of microglia to DAM and how these factors change at different AD stages.

## Physiological functions of TREM2 and pathological effects in AD

**Figure 3:**
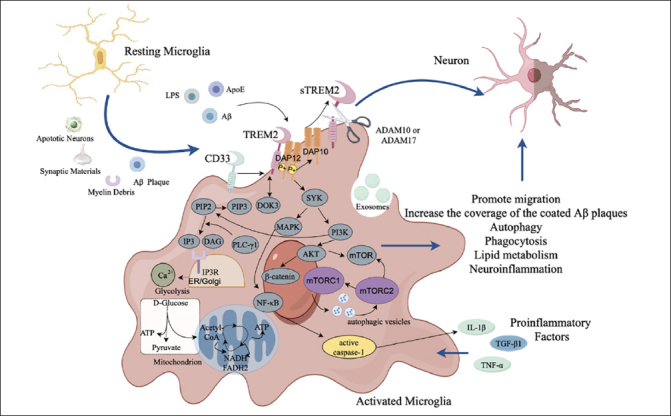
Mechanism of TREM2 affecting microglial function in AD TREM2=myeloid cell trigger receptor 2; sTREM2=soluble TREM2; ApoE=Apolipoprotein E; LPS=lipopolysaccharide; DOK3=downstream of kinase 3; PI=phosphatidylinositol; DAP12= DNAX activation protein 12; DAP10=DNAX activation protein 10; SYK=spleen tyrosine kinase; PI3K=phosphatidylinositol 3-kinase; AKT=RAC-alpha serine/threonine-protein kinase; mTOR=mammalian targets of the rapamycin; mTORC 1=mammalian targets of the rapamycin complex 1; mTORC 2=mammalian targets of the rapamycin 2; NF-κB=nuclear factor κB; MAPK=mitogen activated protein kinase; TLR4=Toll like receptor 4; IL-1β= interleukin-1β; TNF-α=tumor necrosis factor-α; TGF-β1=transforming growth factor-β1; β-catenin=beta-catenin; ATP=adenosine triphosphate; NADH=nicotinamide adenine dinucleotide hydrogen; FADH_2_=Flavin adenine dinucleotide reduced; CD33=cluster of differentiation 33; ADAM10/17=a disintegrin and metalloprotease domain-containing protein 10/17; IP3R=inositol 1,4,5-trisphosphate receptor; PLC-γ = phospholipase c-γ; IP =interferon inducible protein

TREM2 and sTREM2 play important roles on the migration, metabolism, phagocytosis, and inhibition of inflammatory response of microglia. However, many factors might result in expression of TREM2 in microglia, such as apoptotic neurons, myelin debris, and proinflammatory factors. In additional, TREM2 can bind to various ligands, such as LPS, ApoE and Aβ, resulting in signal transduction through transmembrane adaptor protein DAP12/DAP10. The sustained activation of microglia might result in the decline of phagocytosis, dysfunction of lipid metabolism in microglia, and neuroinflammation (Fig.3). Thus, in the pathological states, TREM2 might contribute to the neuronal damage and even AD development.

### TREM2 regulates microglial cell survival, migration, and the barrier effect

TREM2 can affect the survival and migration of microglia. In the in vitro experiments, the absence of TREM2 reduced the survival rate of microglia (60). In addition, DAP10 is an activator of AKT, and the key signaling molecule of extracellular signal kinase inhibits GSK3β activity to promote cell survival and proliferation through PI3K-AKT-GSK3β pathway (46). In an in vivo study on wild-type and TREM2 knockout mice, the sTREM2 triggered microglial activation and promoted survival through PI3K/AKT pathway (32). In additional, the level of CSF sTREM2 shows an AD stage-dependent increase and reaches its peak in the early AD stage, which might be related to the activation and the increase of survival of microglia caused by neuronal damage (61).

TREM2 can recognize Aβ by releasing lipoprotein signals such as ApoE and ApoJ and activates the TREM2-DAP12 signaling pathway, thus promoting microglial migration and aggregation (62). Activated microglia migrate to the vicinity of Aβ plaques by chemokines through FAK/Racl/Cdc42 migration signaling pathway. The migration ability of microglia to Aβ depositions is reduced in microglia with TREM2 R47H mutation (63), which may also be an important cause of AD pathogenesis in TREM2 R47H mutant carriers.

Multiple studies have shown that microglia alleviate the toxicity of Aβ plaques to the neurons through TREM2-mediated the barrier surrounding Aβ plaques, and restricting the spread of Aβ plaques can protect surrounding neurons from the neurotoxicity of the plaques (60, 64). The ability of microglia to coat Aβ plaques was significantly reduced in mice with haploid-deficient either TREM2 or DAP12 and in humans with the TREM2 R47H mutation. Thus, TREM2 deficiency may decrease the ability of microglia to form a neuroprotective microglial barrier in order to encapsulate the Aβ plaque (60). Meanwhile, overexpression of TREM2 increases the coverage and tightness of Aβ plaques encapsulated by microglia, thus reducing the dispersion speed and dispersion degree of Aβ plaques (65). This barrier regulation of Aβ plaque may be a novel therapeutic strategy for AD.

### TREM2 regulates microglial phagocytosis

Microglia participate in the remodeling of the neuronal connectivity network by phagocyting synapses, axons, and myelin fragments, and microglia also maintain CNS homeostasis by directly engulfing bacteria and viruses (66). TREM2 can promote the phagocytosis capacity of microglia through upregulating expression of C/EBPα-dependent CD36 (67). TREM2 and sTREM2 can bind to Aβ, and TREM2 is suggested to be the receptor for Aβ. TREM2 interaction with signaling adaptor DAP12 is enhanced when Aβ interacts with TREM2 (68). After the formation of TREM2-Aβ complex, the phagocytosis of microglia is activated, which mediates the clearance of aggregated and deposited Aβ (34). In turn, enhancing the phagocytosis activity of microglia on Aβ plaques can alleviate the pathological effect of Aβ (32). The level of sTREM2 in CSF might reflect the ability of microglia to clear amyloid plaques (69), which is particularly significant in the early symptom stages of AD.

The microglial phagocytosis on damaged neurons is dependent on microglial TREM2 and neuronal ApoE. ApoE aggregates on the surface of damaged and dead neurons to promote the phagocytosis of neurons by microglia. Compared with in the healthy cells, the level of ApoE on the cell membrane in the apoptotic N2a cells (a mouse neuroblastoma cell line) is significantly increased; the level of phagocytosis of apoptotic N2a cells by microglia is also significantly increased (70). Further, the microglia with TREM2-knockout cannot phagocytose lipidated ApoE, indicating that TREM2 is required for phagocytosis of ApoE and ApoE-mediated phagocytosis on damaged neurons (63).

At present, many researches focus on the regulation of the phagocytic function of microglia themselves, but there is few research that focus on antigen presentation of microglial extracellular vesicles in the immune process. Exosomes are bioactive vesicles produced by intracellular systems. The unique function of microglial exosomes is to act as messenger vesicles for intercellular communication and antigen recognition during microglial phagocytosis (71). The expression of TREM2 on the membrane of exosomes leads to an increase in Ca^2+^ levels in the exosomes of microglia (Ca^2+^ controls the secretion of exosomes but does not affect their size) (72). Exosomes of microglia bind Aβ in a TREM2-dependent manner, which alters the inflammatory environment around senile plaques and promotes the phagocytosis of Aβ plaques by microglia (73).

### TREM2 inhibits neuroinflammation

Neuroinflammation is a protective stress response to prevent brain damage but the sustained neuroinflammation might result in neuronal damage. The imbalance of anti-inflammatory factors and proinflammatory factors can lead to increased neuroinflammation in the brain and cause irreversible damage to the nervous tissue. Studies in vitro have found that TREM2 inhibits the production of inflammatory factors in microglia, thereby alleviating neuronal damage (74). Phosphorylation of tyrosine kinases induced by DAP12 leads to the activation of anti-inflammatory downstream signaling pathways. The upregulation of TREM2 expression decreases the expression of the Toll-like receptor (TLR) family (TLR2, TLR4 and TLR6), suppressing the production of pro-inflammatory cytokines including interleukin-1β (IL-1β), IL-6, and tumor necrosis factor α (TNF-α) (75). Aβ1-42 oligomers promote neuroinflammation and neuronal death in AD brain by promoting microglial release of pro-inflammatory cytokines and by inhibiting production of anti-inflammatory factors such as transforming growth factor-β1 (TGF-β1) (76). TNF-α inhibits the phagocytosis of Aβ by microglia and upregulates γ-secretase activity, thereby promoting Aβ depositions and microglia-mediated neuroinflammation. In additional, the accumulated Aβ can activate the Tolllike receptor 4 (TLR4) (77), which leads to the activation of the MAPK and NF-κB signaling pathways. Indeed, TREM2 is like a double-edged sword. On the one hand, TREM2 prevents proinflammatory responses through the PI3K/NF-κB signal pathway. On the other hand, it enhances the expression of proinflammatory cytokines and type I interferons through the TLR4/NF-κB pathway (78). These factors, such as NF-κB-mediated miRNA-34a, LPS-induced signaling, and pro-inflammatory cytokines, might lead to the downregulation of TREM2 expression (79), whereas anti-inflammatory cytokines, such as IL-4 and IL-13, upregulate TREM2 expression (32). The activation of NF-κB pathway induced by sTREM2 not only leads to the production of inflammatory cytokines but also promotes the survival of microglia through regulating AKT/GSK3β/β-catenin signaling pathway (32). This is consistent with the observation that the increase in CSF sTREM2 of AD patients is associated with an increase in inflammatory process (80). Therefore, sTREM2 is considered as a bait receptor because it can trigger the production of inflammatory cytokines, thereby inducing the transformation and immune response of microglia. Thus, the role of TREM2 in regulating neuroinflammation by microglia and the impact of TREM2 on the pathological progression of AD deserve further attention.

### TREM2 regulates microglial autophagy and glucose metabolism

TREM2 activates mTOR signaling through DAP12/DAP10 to recruit upstream mTOR activators, thereby maintaining microglial metabolism (49). Activation of mTOR leads to the activation of the mammalian targets of the rapamycin complex 1 (mTORC1) and mTORC2 involved in the inhibition of autophagy (81). Mutations in TREM2 cause the aberrant expression of autophagy-related proteins, such as LC3, Beclin1, and p62 (82). This indicates that TREM2 is important for controlling autophagy.

In normal homeostasis, microglia rely primarily on oxidative phosphorylation to produce ATP. When activated, however, microglia might reprogram from oxidative phosphorylation to glycolysis to meet the energy requirements and to make functional responses. The mTOR activates hypoxia inducible factor-1α (HIF1α), which stimulates expression of glycolysis-related genes and regulates the production of ATP (53). In hypoxic environments, HIF1α increases the expression of hexokinase, resulting in more glucose to be phosphorylated and metabolized through the glycolytic pathway. HIF1α can also inhibit mitochondrial electron transport chain complexes and thus reduce mitochondrial oxygen consumption and ATP production. Therefore, microglia will be more dependent on glycolysis to meet the energy needs (83, 84).

A study on PET imaging of microglia with a radioligand of 18 kDa transporter (TSPO) demonstrated that glucose utilization is reduced in the brains of mice with TREM2 T66M mutant (85). The microglia from AD patients with loss-of-function (LOF) variants of TREM2, including R47H, W50C and T66M, were short of oxidative phosphorylation and mitochondrial respiratory capacity (86, 87). The ability on phagocytosis of Aβ1-42 was impaired in the microglia with LOF variants of TREM2 but restored after activation of PPAR-γ, a gliazone receptor involved in glucose metabolism and mitochondrial biogenesis (88). In a recent study on humanized ApoE3 and ApoE4 mice lacking TREM2, the dysregulation of glucose transport and metabolism was also detected. These results indicate that ApoE and TREM2 in microglia are related to each other. Reconstruction of ATP levels using cyclocreatine could improve microglial function both in vitro and in vivo and alleviate the damage of Aβ plaques to adjacent neurons in plaques (71).

In general, TREM2 maintains the basic cellular metabolism and promotes glucose utilization in microglia by maintaining mTOR signaling and metabolic adaptability.

### TREM2 regulates lipid metabolism in microglia

In the CNS, TREM2 affects the metabolisms of cholesterol, myelin sheath, and phospholipids(89). TREM2 can bind lipid-associated ligands, for example, phospholipids (34), high-density lipoprotein (HDL), low-density lipoprotein (LDL), lipids (90) and ApoE (91, 92) contained in the apoptotic neurons, and so on. ApoE mediates the endocytosis and secretion of lipids and cholesterol (93, 94). Furthermore, TREM2 promotes the transition of microglia to DAM through several lipid-associated pathways.

ApoE is the most important lipid transporter protein in the body and has the greatest impact on lipid metabolism, mediating the endocytosis and uptake of lipids and cholesterol. ApoE is involved in AD development. It has been found that ApoE is highly expressed in microglia of AD patients (94). ApoE4 isoform is the AD risk variant and it results in more lipid droplets (LD) to accumulate in microglia. Recent studies have found that LD would accumulated in microglia of aging mice and the microglia represented a dysfunctional and proinflammatory state (called lipid-droplet-accumulating microglia (LDAM) state) (95). The lipid accumulation in microglia induced by ApoE4 weakens the response of microglia to neuronal activity and thus disrupting the coordinated activity of neuronal populations (15). Therefore, the ApoE4 genotype promotes the transition of microglia to an evolutionarily conserved, maladaptive, and destructive LDAM state. Further, Aβ induces the synthesis of triglyceride lipid, LD accumulation, and subsequent secretion of neurotoxic factors in microglia in an ApoE dependent manner. These lipids may be transferred to neurons, thereby inducing neurodegeneration. The elevated level of sTREM2 in the cerebrospinal fluid of AD patients is also positively correlated with neuroinflammatory markers, microglial dysfunction, and neuronal damage. In addition, the assays of unbiased transcriptomics and lipidomics screening indicated that sTREM2 leads to the excessive activation of microglia, inhibition of phagocytosis on myelin debris and lipid metabolism, and enhancement of glycolysis through NF-κB pathway (96).

#### TREM2 regulates brain cholesterol and myelin metabolism

Many myelin and brain cholesterol are both ligands for TREM2, and thus TREM2 can also mediate myelin phagocytosis (70, 97, 98). TREM2 deficiency was recently found to be associated with the efflux defect of microglial cholesterol, resulting in increase of intracellular cholesterol ester (CE) (57). The coding variation of PLCG2, a gene encoding the microglial phospholipase, resulted in a significant increase in lipids such as CE, free cholesterol, ceramide, thiolate, phospholipids, triacylglycerol, and DAG. TREM2 regulates cholesterol transport in microglia in a PLCγ2-dependent manner. The blood-brain barrier (BBB) prevents cholesterol-rich lipoproteins from entering the CNS, and 80% of free cholesterol in brain exists in the myelin sheath, which is formed by oligodendrocytes to isolate axons. Thus, myelin is an important and sensitive marker of cholesterol metabolism in brain (99). It has been shown that TREM2 participates in the microglial response to myelin injury and thus affects remyelination (100). TREM2 plays a protective role in microglia in response to myelin injury, and it may influence the transcriptional program of microglia to enhance lipid metabolism and myelin debris clearance, thereby promoting myelin regeneration. On the contrary, higher levels of sTREM2 in CSF results in the abnormality of lipid accumulation, cholesterol turnover, and glycolysis through NF-κB signaling pathway, which is involved into the excessive phagocytosis of myelin debris and changes in immune metabolism of microglia (96).

### TREM2 binds to phospholipids to regulate phospholipid metabolism

TREM2 binds to injury-associated phospholipids and acts as a scavenger receptor for apoptotic neurons that occur during neuronal injury. In the brain of AD mice, large amounts of phosphatidylserine (PS) and phosphatidylethanolamine (PE) were exposed to synaptosomes to induce TREM2-mediated signaling (35). Downstream of kinase 3 (DOK3) is a cohesive protein with physical interaction with TREM2 and DAP12. TREM2 interferes with phosphatidylinositol (PI) metabolism by interacting with DOK3. Thus, the level of PIP2 in the plasma membrane may be increased by the inhibition of PLCγ2 activity when TREM2 mutations lead to the activation of DAP12. In contrast, the inhibition of TREM2 is beneficial against AD (101).

### TREM2 is related to Aβ and tau pathologies

TREM2 and AD pathologies including Aβ and tau may interact with each other. On the one hand, TREM2 can act as an upstream regulator of Aβ, regulating the morphology and toxicity of Aβ plaques. The deposited Aβ triggers tau hyperphosphorylation and further aggregation to form NFT, promoting neuronal damage and synaptic loss and ultimately leading to cognitive impairment. On the other hand, the oligomeric Aβ might bind to TREM2 and further regulate the microglial activity, even resulting in the conversion from normal microglia to disease-associated microglia. The pathological examination on the human brain showed that microglia exhibited morphological degeneration earlier than tau pathology, which may be caused by microglial aging or chronic Aβ toxicity (102–104).

#### TREM2 and Aβ pathology

First, the multiple studies have demonstrated that microglia regulate Aβ plaques toxicity through TREM2 and a barrier around Aβ plaques. Restricting the spread of plaques can protect the neurons surrounding plaques from the neurotoxicity of Aβ (60, 64). In the *in vitro* experiments, the absence of TREM2 reduces the microglial survival and affects the binding of microglia to Aβ plaques. It leads to a significant decrease in plaque coverage with microglia and to an increase in more toxic filamentous plaques, and it also exacerbates malnutrition of neurites surrounding the plaques (63). In the *in vivo* study of the 5 x FAD mice model also showed that TREM2 deficiency resulted in the failure of microglia to cluster around Aβ plaques and the microglial apoptosis, accompanying the augment of Aβ accumulation (34). In addition, microglia deficient in the SYK, a downstream of TREM2-DAP12/DAP10 signaling pathway, similarly failed to encase Aβ plaques and accelerated brain pathology and behavioral deficits through the impairment of the PI3K-AKT-GSK3β-mTOR pathway (105).

Secondly, TREM2 plays a role in regulating the expression of AD-related genes in microglia, which might involve Aβ pathology. When TREM2 is deficient, the microglial gene expression profile in 5 x FAD mice is highly silenced (34). However, in BAC-TREM2 mice (expressing human TREM2 in microglia), the composition of Aβ plaques was shifted significantly to less filamentous and more inert forms. The levels of both soluble and insoluble Aβ_1–42_ were significantly reduced in BAC-TREM2 mice (106). Implantation of bone marine-derived mesenchymal stem/stromal cells (MSCs) with overexpression of TREM2 into the brains of APP/PS1 mice also reduced Aβ production and deposition (107). In spatial, DAM is correlated with Aβ plaques and it might swallow Aβ particles. In AD process, DAM display the shifts of gene expression, downregulating the levels of several microglial homeostatic genes including P2ry12/P2ry13, Cx3cr1, and Tmem119 (108). Concurrently, a subset of genes within DAM are upregulated, including the AD risk factors like ApoE, Ctsd, Tyrobp, and Trem2 (109).

In general, in the role of TREM2, microglia exert neuroprotective effects in early AD by inhibiting the more toxic filamentous Aβ, suppressing the aggregation and diffusion of Aβ, and silencing the expression of AD related genes.

#### TREM2 and tau pathology

Increasing evidence supports that TREM2 signaling is related to pathological tau. In the *in vivo* experiments, The TREM2 knockout decreases the response of microglia to pathological tau (110); tau aggregation and diffusion as well as increased neuritic plaque were found in a TREM2-knocked out APP/PS1 mice model (111). Upregulation of TREM2 can inhibit the activity of GSK3β by activating the PI3K/Akt signaling pathway, thereby inhibiting the phosphorylation of tau protein in both APP/PS1 mice and cultured SH-SY5Y cells (112). Additionally, silencing TREM2 in the brains of P301S mice significantly increases the activity of GSK3β and CDK5, both of which are important factors in tau hyperphosphorylation (113, 114).

Although TREM2 has been suggested to link to AD, its role in regulating intracellular tau pathology remains controversial. TREM2 might be located at a critical intersection of Aβ and tau pathology (111). On one hand, in the presence of Aβ pathology only, the accumulation and spread of tau will be also aggravated, resulting in neurodegeneration. On the other hand, the study with tau APP/PS1 mice model with TREM2 knockout found that tau aggregation in the mice brain of early (9 months) and late (17 months) was significantly increased, but tau aggregation in tau-P301L mice with TREM2 knockout was not affected. Therefore, it implies that TREM2 specifically inhibits Aβ-driven tau pathology (115). Therefore, more investigations are needed to clarify the role of TREM2 in tau pathology.

## Agonistic antibodies of TREM2 for AD treatment

The strategy targeting for TREM2 is an alternative method for AD therapy. At present, most of AD drugs were developed through directly targeting for pathological features such as amyloid protein deposition, abnormal tau proteins, or the cholinergic system. AD is a disease involving multiple pathological factors, and the drugs targeting a single pathological factor might not well prevent the occurrence and development of this diseases. Recently, TREM2 agonistic antibody have been suggested as competitive candidate for AD treatment. These antibodies have the potential advantage over small molecules, for example, TREM2 agonistic antibodies can bind the specific sites within TREM2 without penetrating the cell, thus avoiding unpredictable side effects (116). Some TREM2 agonistic antibodies have been developed (Tab. 1).

**Table 1:** The protective TREM2 agonistic antibodies

**ANTIBODY**	**SPECIES**	**THE MECHANISM OF ACTION**	**MOUSE MODEL OF ALZHEIMER’S DISEASE (AGE)**	**ANTIBODY ACTION ON MICROGLIA**	**ANTIBODY ACTION IN AD**	**COGNITIVE ABILITY**	**REFERENCE**
4D9	mouse	Having a stem region epitope close to the cleavage site, by stabilizing TREM2 on the cell surface and reducing its shedding while activating phospho-SYK signaling	APP-NL-G-F (6 months)	Stimulate the survival of macrophages and increase the phagocytic effect of microglia	Reduced soluble amyloid β	Did not report	Schlepckow et al (2020) (117)
AL002c	human	Did not report	5 × FAD, hTREM2-BAC (5–5.5 months)	drives metabolic activation and proliferation of microglia in vivo	Reduced diffuse plaques; reduce inflammatory response.	Enhanced	Wang, et al. (2020)(118)
Ab-T1	human and mouse	sTREM2	5×FAD (4–5 months)	activates microglia	reduce tau burden; alleviate the inflammatory response	Enhanced	Fassler et al. (2021) (125)
T2AB	human	Did not report	5 × FAD, hTREM2-BAC (5 months)	Induced a shift in gene expression patterns in microglia	No change in soluble or insoluble amyloid β	Did not report	Ellwanger et al (2021) (100)
A18b	mouse	Increase TREM2 aggregation	5 × FAD (at 5 months)	Enhance the phagocytic effect of microglia; Enhance migration towards Aβ plaques	reduced endogenous tau hyperphosphorylation	Enhanced	Zhao et al. (2022)(121)
Ab 2	human	targeting the transferrin receptor (TfR)	5 × FAD, human microglia transplanted into the lateral ventricle (5 months)	Enhance the phagocytic effect of microglia	Enhance the interaction between microglia and plaques	Did not report	Zhao et al. (2022)(126)
AL002a	mouse	promote TREM2-dependent DAP12 and SYK phosphorylation activation	5 × FAD, injected Alzheimer’s tau aggregate injected from human brain tissue (6 months)	Promote the survival and proliferation of microglia	Exacerbate the seeding and spread of pathological tau; Neuritic dystrophy	Did not report	Jain, et al. (2023) (127), respectively
DNL919	human being	targeting TfR	5 × FAD, hTREM2-BAC (4–5 months)	Promote the survival and proliferation of microglia	Did not report	Did not report	Van Lengerich et al (2023) (124)
ATV:TREM2	human being	targeting TfR	Did not report	Enhance the activity of microglia; Improve glucose metabolism	Promoted microglia activity and glucose metabolism	Enhanced	Michael Fassler et al. (2023)(128)

### Agonistic antibody against Aβ and tau pathologies

The TREM2 agonistic antibody can block the cleavage of TREM2 in the stem region and prevents its shedding from microglia, thereby enhancing the ability of microglia to phagocytose Aβ and neuronal debris. Therefore, TREM2 agonistic antibody might reduce the burden of amyloid plaques and increase the aggregation of microglia around amyloid plaques. The first agonistic antibody of TREM2 is the monoclonal antibody 4D9, which shows the ability to reduce the amyloid plaques in AD mouse models (117). The 4D9 antibody binds to an epitope located 12 amino acids away from the ADAM 10 and ADAM 17 cleavage site. It activates downstream SYK signaling and strongly increases rat macrophage survival through binding full-length TREM2 on the cell surface with high affinity and thus inhibiting ADAM 10 or ADAM 17 hydrolytic cleavage of TREM2 in a dose-dependent manner (117). In addition, the humanized monoclonal antibody AL002 is a TREM2 agonistic antibody developed by Alector company. Researches are ongoing to evaluate its effect in improving the symptoms and pathology of AD. AL002 has been used to confirm that the activated TREM2 reduces Aβ amyloid plaques in the brain (118, 119). These results provide early evidence for the follow-up study of TREM2 agonistic antibodies.

In additional, the agonistic antibodies might also enhance the intensity of synaptic and neuronal marker, reduce endogenous tau hyperphosphorylation, and improve cognitive impairment in tested AD mice (120, 121).

### Clinical trials using TREM2 agonists and clinical perspectives

There are limited clinical trial data available for TREM2 agonistic therapy. The results of a phase I clinical trial showed that the TREM2 agonistic antibody AL002 was safe and well tolerated among 69 participants. Currently, the phase II trial of AL002 is underway to evaluate the efficacy in patients with mild cognitive impairment and mild Alzheimer’s disease dementia (118). The research based on biomarkers suggests that the beneficial effects of TREM2 agonistic antibody may be related to the stage of AD development.

One of the biggest challenges of TREM2 agonistic antibody therapy is that systemic antibody administration requires very high doses to achieve treatment-relevant antibody concentrations in the brain (122). Usually, only about 0.1% of administered antibodies cross the blood-brain barrier and enter the brain parenchyma (123). Therefore, it is crucial to enhance the effect of antibodies crossing the blood-brain barrier. Recently, many antibodies have been designed with a binding site of monovalent transferrin receptor (TfR) to facilitate the transport of the antibody from the blood into the brain (124). It may be a potential strategy to enhance the bioavailability of TREM2 agonistic antibody.

## Conclusion and perspectives

Many studies have been conducted on how TREM2 affects the process of AD pathology, and many important results have been achieved. There are a wide variety of TREM2 ligands and complex signaling networks, and further studies are needed to explain how TREM2 signaling regulates physiological function and pathological effects of microglia in the context of a complex AD.

With the rise and deepening of TREM2 research in AD development, there is a glimmer of hope for the treatment based on the pathogenesis of AD. The discovery and the use of therapeutic drugs for AD treatment in clinical are impacted by many factors. At present, there is no specific drug that directly targets Aβ accumulation except for Aβ antibody in clinic. The treatment with agonistic antibody of TREM2 could be a novel approach for the AD treatment. Although the protective effects of TREM2 agonistic antibody is becoming increasingly apparent, there are still several issues. The long-term research is needed not only to address the improvement phenotype of AD treatment but also to consider potential harmful effects, such as an increased risk of tumor development. We still need to better understand the function of sTREM2, as almost all therapeutic antibodies studied so far not only bind to full-length TREM2, but also to sTREM2. Therefore, further research on the mechanism of TREM2 and sTREM2 on microglial function will help to develop the effective therapeutic strategies for the onset stage of AD and to promote the large-scale clinical trials, which may have important theoretical value and practical significance for the treatment of AD.
